# NSF Fellows’ perceptions about incentives, research misconduct, and scientific integrity in STEM academia

**DOI:** 10.1038/s41598-023-32445-3

**Published:** 2023-04-07

**Authors:** Siddhartha Roy, Marc A. Edwards

**Affiliations:** 1 Department of Civil and Environmental Engineering, Virginia Tech, 1145 Perry St., 418 Durham Hall, Blacksburg, VA 24061 USA; 2 The Water Institute at UNC, Gillings School of Global Public Health, University of North Carolina at Chapel Hill, Chapel Hill, NC 27516 USA

**Keywords:** Human behaviour, Computer science, Engineering, Civil engineering

## Abstract

There is increased concern about perverse incentives, quantitative performance metrics, and hyper-competition for funding and faculty positions in US academia. Recipients of the prestigious National Science Foundation Graduate Research Fellowships (n = 244) from Civil and Environmental Engineering (45.5%) and Computer Science and Engineering (54.5%) were anonymously surveyed to create a baseline snapshot of their perceptions, behaviors and experiences. NSF Fellows ranked scientific advancement as the top metric for evaluating academics followed by publishing in high-impact journals, social impact of research, and publication/citation counts. The self-reported rate of academic cheating was 16.7% and of research misconduct was 3.7%. Thirty-one percent of fellows reported direct knowledge of graduate peers cheating, and 11.9% had knowledge of research misconduct by colleagues. Only 30.7% said they would report suspected misconduct. A majority of fellows (55.3%) felt that mandatory ethics trainings left them unprepared for dealing with ethical issues. Fellows stated academic freedom, flexible schedules and opportunity to mentor students were the most positive aspects of academia, whereas pressures for funding, publication, and tenure were cited as the most negative aspects. These data may be useful in considering how to better prepare STEM graduate trainees for academic careers.

## Introduction

The U.S. scientific enterprise grew exponentially in the post-World War II era with large financial investments from the federal government. Several high-profile cases of alleged research misconduct in the 1980s forced Congress to push for legislative oversight, ombuds offices at funding institutions, and university protocols to address unethical behavior^1–3^. In the twenty-first century, there are concerns that the rising importance of possible perverse incentives (i.e., emphasizing quantitative performance metrics, funding, high impact journal publications and prestige) in STEM academia might undermine the quality of research performed, maintenance of high ethical standards and productive use of taxpayer dollars^4–6^. For example, it is hypothesized that rewarding researchers for higher publication or citation counts can lead to “natural selection” of substandard science and reduced emphasis on quality hypotheses and research questions^7–9^. Further, as journal retractions rise^10^, it remains unclear the extent to which science is self-correcting, and this trend has been variously attributed to pressures to publish and garner funding, misconduct policies, academic culture and investigator career stage^11,12^. Maintaining scientific integrity is deemed “vital” for the US’ national interest^13^, but there is relatively little data on this subject from targeted surveys of high-performing US researchers.

In this article, we report results from an anonymous, online survey of U.S. National Science Foundation (NSF) Graduate Research Fellowship recipients (hereafter, “NSF Fellows” or “Fellows”) on their perceptions of STEM and academia. The survey posed questions on cheating, research misconduct, formal integrity training and ethical environments, as well as the overall positives and negatives of academia^5,14,15^. NSF’s definition of research misconduct, i.e., the “willful fabrication, falsification, plagiarism, and other questionable practices,” was displayed before survey respondents answered questions on the topic^16^. The survey was administered in February–May 2019 before the COVID-19 global pandemic and targeted Fellows from Civil and Environmental Engineering (CEE) or Computer Science and Engineering (CSE). These were selected as two broad STEM disciplines that have transformed society but currently face concerns about ethics^17–19^ and high competition for faculty positions^20^.

## Methods

This study was approved by Virginia Tech’s Institutional Review Board (IRB #17-677) and administered online via Qualtrics (www.qualtrics.com) between February 18–May 02, 2019. The names, baccalaureate institutions, and proposed fields of study of individuals first receiving their NSF Graduate Research Fellowships during 2002–2007 and 2012–2017 in CSE and CEE disciplines (n = 1662) were downloaded from www.nsfgrfp.org. Active email addresses could be retrieved for 1078 fellows through online searches, who were each sent one recruitment and one reminder email containing a unique survey link. All Fellows read an electronic informed consent form before agreeing to participate. Fellows completing the survey received US$25 Amazon.com electronic gift cards through a department Amazon account not tied to the study investigators. Given the sensitive nature of some questions, Fellows were assured anonymity through Qualtrics’ "Anonymize Response" setting, which decouples survey responses from respondent email addresses. Incomplete survey responses were not included in the final dataset and analyses.

All data were analyzed and graphed in Minitab 19.2020.1, Microsoft Excel 2016, QDA Miner Lite 2.0.6, and Datawrapper (www.datawrapper.de). Summary statistics tables and cross-tabulations were generated in Minitab. Fisher’s exact test of independence and Pearson’s Chi-Square test were used to analyze cheating and misconduct responses by NSF Fellows’ gender, cohort year, discipline and academic stage. A p-value below 0.05 was used to establish statistical significance for assigned variables. The responses could be classified by discipline for 218 of 244 (89.3%) Fellows based on primary undergraduate major, dates respondents began filling out the survey (recruitment emails were sent to CEE and CSE email lists during different weeks) and responses to open-ended questions mentioning discipline. All methods, including the coding and categorization of qualitative responses to open-ended questions, were conducted in accordance with relevant guidelines and methods for qualitative data analysis^21^ in Excel and QDA Miner Lite. Specifically, survey responses were assigned categories, and these categories were systematically reorganized and merged into larger and more representative themes in later iterations. A subset of Fellows’ responses was also extracted and reproduced as-is in text to properly contextualize the themes.

## Results

### Survey demographics

The overall response rate for this survey was 22.6%, based on 1078 Fellows who received recruitment emails (Tables S1 and S2) containing unique links to the survey (Text S1). While survey email delivery rates were comparable for CSE And CEE disciplines (62–66.5%), the response rates were higher in CEE (30.2%) over CSE (18.7%). It is unclear why the response rates differ. As one survey eligibility criterion was that every respondent should have received formal ethics training, this may explain the somewhat low overall participation rates. The respondent pool (n = 244) was relatively evenly distributed between the CSE (54.5%; n = 133) and CEE (45.5%; n = 111) disciplines, and between female (50.8%, n = 124) and male (48.4%, n = 118) genders. Eighty-one percent (n = 198) were awarded their fellowships in 2012–2017 and the rest (18.9%; n = 46) in 2002–2007 (Table 1). Ninety-four percent (94.3%; n = 230) finished their undergraduate studies with one major, while 5.7% (n = 14) had 2–3 majors, with 32.8% (n = 80) majoring in Computer Science, and 32.4% (n = 79) majoring in Civil Engineering, Environmental Engineering or Environmental Science. Fifty-six percent (56.1%; n = 137) were enrolled in graduate school when they took the survey, while the remaining 43.9% (n = 107) had graduated. A majority of NSF Fellows were employed in academia as graduate students, postdocs or untenured faculty (50.8%) and tenured/tenure-track professors (20.1%).

**Table 1:** Demographics.

Category	Response	Count (%)
Subject area (sub-disciplines in Table S2)	Civil and Environmental Engineering	111 (45.5)
	Computer Science and Engineering	133 (54.5)
GRFP Cohort year	2002–2007	46 (18.9)
	2012–2017	198 (81.1)
Gender	Male	118 (48.4)
	Female	124 (50.8)
	Others: “Female (Trans woman)”, “Non-binary (assigned Female at birth)”	2 (0.8)
Undergraduate major(s)*	Civil Engineering or Environmental Engineering or Environmental Science	80
	Computer Science	79
	Electrical or Computer Engineering	25
	Mathematics	14
	Mechanical Engineering	8
	Chemical or Mining or Metallurgical Engineering	6
	Others	47
Respondent status (in Spring 2019)	MS student	4 (1.6)
	PhD student (post-MS)	60 (24.6)
	PhD student (direct)	73 (29.9)
	**Total students**	**137 (56.1)**
	Working post-MS	6 (2.5)
	Working post-PhD	101 (41.4)
	**Total working**	**107 (43.9)**
Current area of employment	Academia (Graduate Student/Postdoc/ Researcher/Non-tenured)	124 (50.8)
	Academia (Tenure-track/Tenured)	49 (20.1)
	Industry/Consulting/Startup/Entrepreneur	42 (17.2)
	Nonprofit/Government	16 (6.6)
	Research Laboratory	11 (4.5)
	Others *(“unemployed”—CEE; “unsure”—CSE)*	2 (1.6)
Considering or pursuing a research career?	Yes	195 (79.9)
	No	49 (20.1)

Summary of NSF Fellow respondents on this survey.

*Total # of undergraduate majors reported exceeds total # of NSF fellows (244), because 14 fellows reported at least two majors (one fellow reported three).

### Research evaluation criteria and academia pros vs. cons

**Figure 1:**
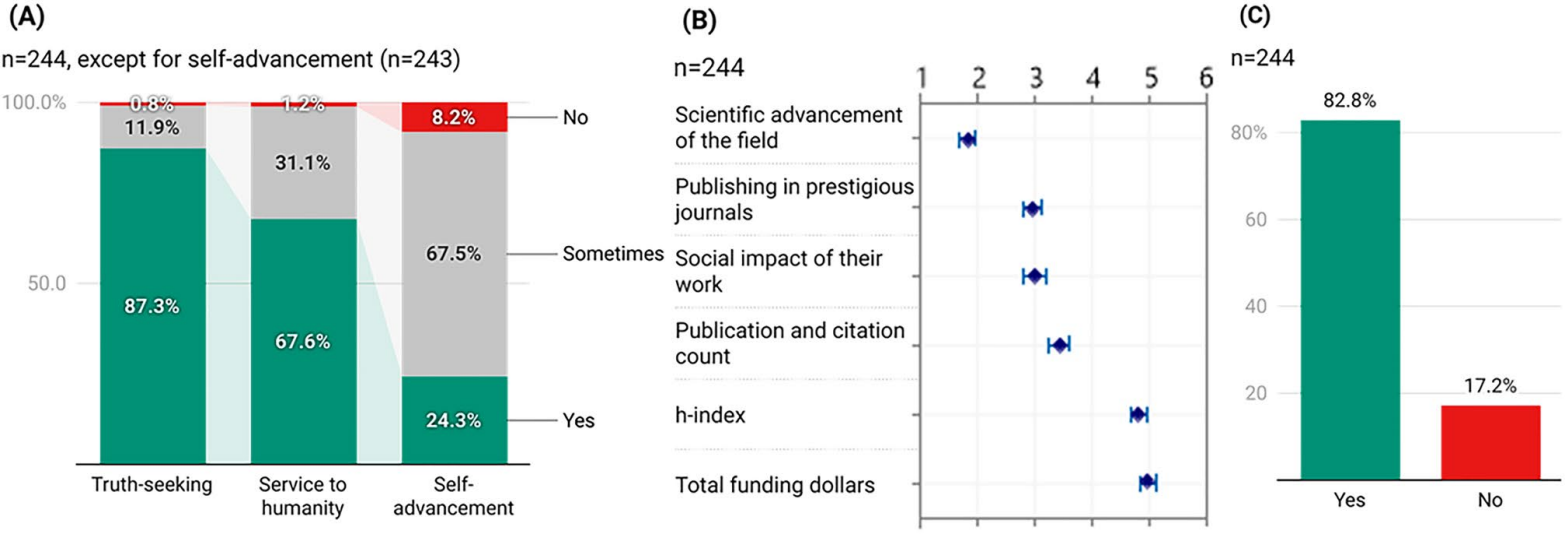
Science and scientists. (**A**) NSF Fellows who believe research is or should be about truth-seeking, service to humanity, or self-advancement. (**B**) Criteria Fellows use to evaluate academic peers (ranked in decreasing order of importance). (**C**) Whether Fellows applied the same criteria for themselves?

A majority of Fellows agreed that research ideally is or should be about truth-seeking (87.3%; n = 213) and service to humanity (67.6%; n = 165) (Fig. 1A). In contrast, when asked about self-advancement only 24.3% (n = 59) felt that should be a primary objective while 67.5% stated it should sometimes be an objective. Only 8.2% (n = 20) said research should not be about self-advancement. On a Likert scale, NSF Fellows arranged six criteria they used to evaluate their peers from most (1) to least important (6) and the results (Fig. 1B) in order of decreasing importance was (1) [highest ranked] scientific advancement of their field (µ = 1.81, σ = 1.23), (2) publishing in prestigious journals (µ = 2.95, σ = 1.21), (3) social impact of their work (µ = 3.00, σ = 1.6), (4) publication and citation count (µ = 3.43, σ = 1.38), (5) h-index (µ = 4.83, σ = 1.21), and (6) total funding procured (µ = 4.98, σ = 1.12). About 8 out of 10 Fellows (82.8%; n = 202) said they used the same metrics to evaluate their own academic careers (Fig. 1C).

In response to open-ended questions asking Fellows to list the most positive aspects of academic life (Table 2), the following were ranked in order of decreasing importance: (1) academic freedom (59%; n = 144), (2) flexible schedule (33.6%; n = 82), (3) opportunity to mentor students (29.1%; n = 71), (4) intellectually stimulating work (22.1%; n = 54), (5) teaching courses (21.3%; n = 52), and (6) autonomy over their careers (13.5%; n = 33). The top cited negative aspects (Table 2) were (1) time spent in constantly writing grants (27.9%; n = 68) or excessive workload or long hours (27.9%; n = 68), (3) high stress or pressures (24.6%; n = 60), (4) low salary (24.2%; n = 59), (5) pressure to publish (22.1%; n = 54), 6) poor work-life balance (19.7%; n = 24) and (7) hyper-competition (18.4%; n = 45).

**Table 2:** Top 10 pros and cons of STEM academia.

Pros (Rank)	Count (%)	Cons (Rank)	Count (%)
Academic freedom (1)	144 (59%)	Constant writing of grants (1)	68 (27.9%)
Flexible schedule (2)	82 (33.6%)	Excessive workload or long hours (1)	68 (27.9%)
Mentoring students (3)	71 (29.1%)	High stress and pressure (2)	60 (24.6%)
Intellectually stimulating work (4)	54 (22.1%)	Low pay (3)	59 (24.2%)
Teaching (5)	52 (21.3%)	Pressure to publish (4)	54 (22.1%)
Autonomy (6)	33 (13.5%)	Poor work-life balance (5)	48 (19.7%)
Intellectual environment or lifelong learning (7)	30 (12.3%)	Hyper-competition (6)	45 (18.4%)
Benefit society or make a difference (8)	27 (11.1%)	Extreme pressure to get tenure (7)	36 (14.8%)
Job security (9)	26 (10.7%)	Institutional politics (8)	19 (7.8%)
Create new knowledge (10)	20 (8.2%)	Limited job prospects or location inflexibility (9)	16 (6.6%)
Prestige or respect (10)	20 (8.2%)	Excessive bureaucracy (10)	12 (4.9%)

Ranked in decreasing order by count and percentage.

### Academic dishonesty

Sixteen percent of Fellows (16%, n = 39) self-reported cheating in graduate school (male = 16.9%; female = 14.5%). The 2002–2007 cohort reported an 8.7% incidence whereas the 2012–2017 cohort reported a 17.7% incidence. Finally, the cheating rate for CEE (23.8%) was significantly higher than that for CSE (11.1%) (Table 3 and S3). Thirty-one percent (31.1%; n = 76) reported having seen their graduate peers cheat (male = 29.7%; female = 33.8%). The rates of NSF Fellows’ knowledge of graduate peers cheating among the 2002–2007 cohort (19.6%) was insignificantly lower than the 2012–2017 cohort (33.8%), whereas that for CEE Fellows (39.6%) was significantly higher than CSE Fellows (23.1%).

**Table 3:** Academic cheating and research misconduct.

No	Summarized prompt or question	Choice	All respondents N (%) 244 (100)	By gender				By award year		
				Male n (%) 118 (100)	Female n (%) 124 (100)	Other n (%) 02 (100)	p-value (M vs. F only)	2002–2007 n (%) 46 (100)	2012–2017 n (%) 198 (100)	p-value
Perceptions and practice of academic cheating in graduate school										
1	I have seen fellow students cheat in graduate classes	Yes	76 (31.1)	35 (29.7)	41 (33.1)	0 (0)	> 0.05**	9 (19.6)	67 (33.8)	> 0.05**
		No	168 (68.9)	83 (70.3)	83 (66.9)	2 (100)		37 (80.4)	131 (66.2)	
2	I have personally cheated in college and/or graduate school	Yes	39 (16)	20 (16.9)	18 (14.5)	1 (50)	> 0.05**	4 (8.7)	35 (17.7)	> 0.05**
		No	205 (84)	98 (83.1)	106 (85.5)	1 (50)		42 (91.3)	163 (82.3)	
3	Maintaining my integrity outweighs incentives to not cheat in class	Yes	217 (88.9)	104 (88.1)	111 (89.5)	2 (100)	> 0.05**	40 (87)	177 (89.4)	> 0.05**
		No	27 (11.1)	14 (11.9)	13 (10.5)	0 (0)		6 (13)	21 (10.6)	
4	I have seen class environments and situations, where cheating was arguably necessary or justified	Yes	57 (23.4)	31 (26.3)	25 (20.2)	1 (50)	> 0.05**	8 (17.4)	49 (24.7)	> 0.05**
		No	187 (76.6)	87 (73.7)	99 (79.8)	1 (50)		38 (82.6)	149 (75.3)	
5	It is possible to create environments^^^ in classrooms where cheating is justified or acceptable	Yes	152 (62.3)	78 (66.1)	73 (58.9)	1 (50)	> 0.05**	20 (43.5)	132 (66.7)	0.0042 **
		No	92 (37.7)	40 (33.9)	51 (41.1)	1 (50)		26 (56.5)	66 (33.3)	
6	The level of cheating I witnessed or engaged in made me think twice about my career choice or the type of people my profession was attracting	Yes	52 (21.3)	24 (20.3)	27 (21.8)	1 (50)	> 0.05**	5 (10.9)	47 (23.7)	> 0.05**
		No	192 (78.7)	94 (79.7)	97 (78.2)	1 (50)		41 (89.1)	151 (76.3)	
Awareness, direct knowledge of and personal involvement in research misconduct in last 5 years										
7	I have heard of research misconduct in my field	Yes	89 (36.5)	46 (39)	42 (33.9)	1 (50)	> 0.05**	17 (37)	72 (36.4)	> 0.05**
		No	155 (63.5)	72 (61)	82 (66.1)	1 (50)		29 (63)	126 (63.6)	
8	I have direct knowledge of research misconduct in my research group, department or field	Yes	29 (11.9)	18 (15.3)	11 (8.9)	0 (0)	> 0.05**	5 (10.9)	24 (12.1)	> 0.05**
		No	215 (88.1)	100 (84.7)	113 (91.1)	2 (100)		41 (89.1)	174 (87.9)	
9	I have personally participated in research involving misconduct, even if I did not commit them myself	Yes	9 (3.7)	5 (4.2)	4 (3.2)	0 (0)	> 0.05 ***	1 (2.2)	8 (4)	> 0.05 ***
		No	217 (88.9)	105 (89)	110 (88.7)	2 (100)		44 (95.7)	173 (87.4)	
		Not sure	18 (7.4)	8 (6.8)	10 (8.1)	0 (0)		1 (2.2)	17 (8.6)	
Propensity to commit or report misconduct										
10	If I felt pressured by my academic advisor or mentor to engage in misconduct^#^, I would do it	Yes	18 (7.4)	9 (7.6)	9 (7.3)	0 (0)	> 0.05 ***	2 (4.4)	16 (8)	> 0.05 ***
		No	139 (57)	69 (58.5)	70 56.5)	0 (0)		31 (67.4)	108 (54.6)	
		I don’t know	87 (35.6)	40 (33.9)	45 (36.3)	2 (100)		13 (28.3)	74 (37.4)	
11	If fabricating and/or falsifying data helped increase my chances to get research funding, scholarship money or publication in a high impact journal, I would do it	Yes	1 (0.4)	1 (0.9)	0 (0)	0 (0)	–	0 (0)	1 (0.5)	–
		No	217 (88.9)	100 (84.7)	115 (92.7)	2 (100)		42 (91.3)	175 (88.4)	
		I don’t know	26 (10.7)	17 (14.4)	9 (7.3)	0 (0)		4 (8.7)	22 (11.1)	
12	If I suspected a researcher of engaging in misconduct, I would report it	Yes	75 (30.7)	34 (28.8)	41 (33.1)	0 (0)	0.0309 *** *X*^*2*^ = **6.956**	15 (32.6)	60 (30.3)	> 0.05 ***
		No	21 (8.6)	16 (13.6)	5 (4)	0 (0)		1 (2.2)	20 (10.1)	
		I don’t know	148 (60.7)	68 (57.6)	78 (62.9)	2 (100)		30 (65.2)	118 (59.6)	
13	Do you feel empowered to raise questions about wrongdoing (however big or small) in your research group?	Yes	190 (77.9)	97 (82.2)	92 (74.2)	1 (50)	> 0.05**	38 (82.6)	152 (76.8)	> 0.05**
		No	54 (22.1)	21 (17.8)	32 (25.8)	1 (50)		8 (17.4)	46 (23.2)	
14	Is “doing the right thing” even though it might negatively impact your career or how you are viewed by peers, something you feel strongly about?	Yes	218 (89.4)	100 (84.7)	116 (93.6)	2 (100)	> 0.05 ***	44 (95.7)	174 (87.9)	–
		No	13 (5.3)	10 (8.5)	3 (2.4)	0 (0)		2 (4.4)	11 (5.6)	
		I don’t care	13 (5.3)	8 (6.8)	5 (4)	0 (0)		0 (0)	13 (6.6)	

NSF Fellows’ perceptions and practice of academic cheating in graduate school (Q1–6), awareness, direct knowledge of and personal involvement in research misconduct in last five years (Q7–9), and propensity to commit or report misconduct (Q10–14).

Percentages may not add up to 100 because of rounding.

^^^e.g., poor design of classes, hypercompetitive grading, unfair homework practices.

^#^Examples given in prompt: “everyone is doing it this way”, “it is okay to throw out bad data”, or publishing results before you are personally confident.

**Fisher’s Exact Test; ***Chi-Square Test.

**Figure 2:**
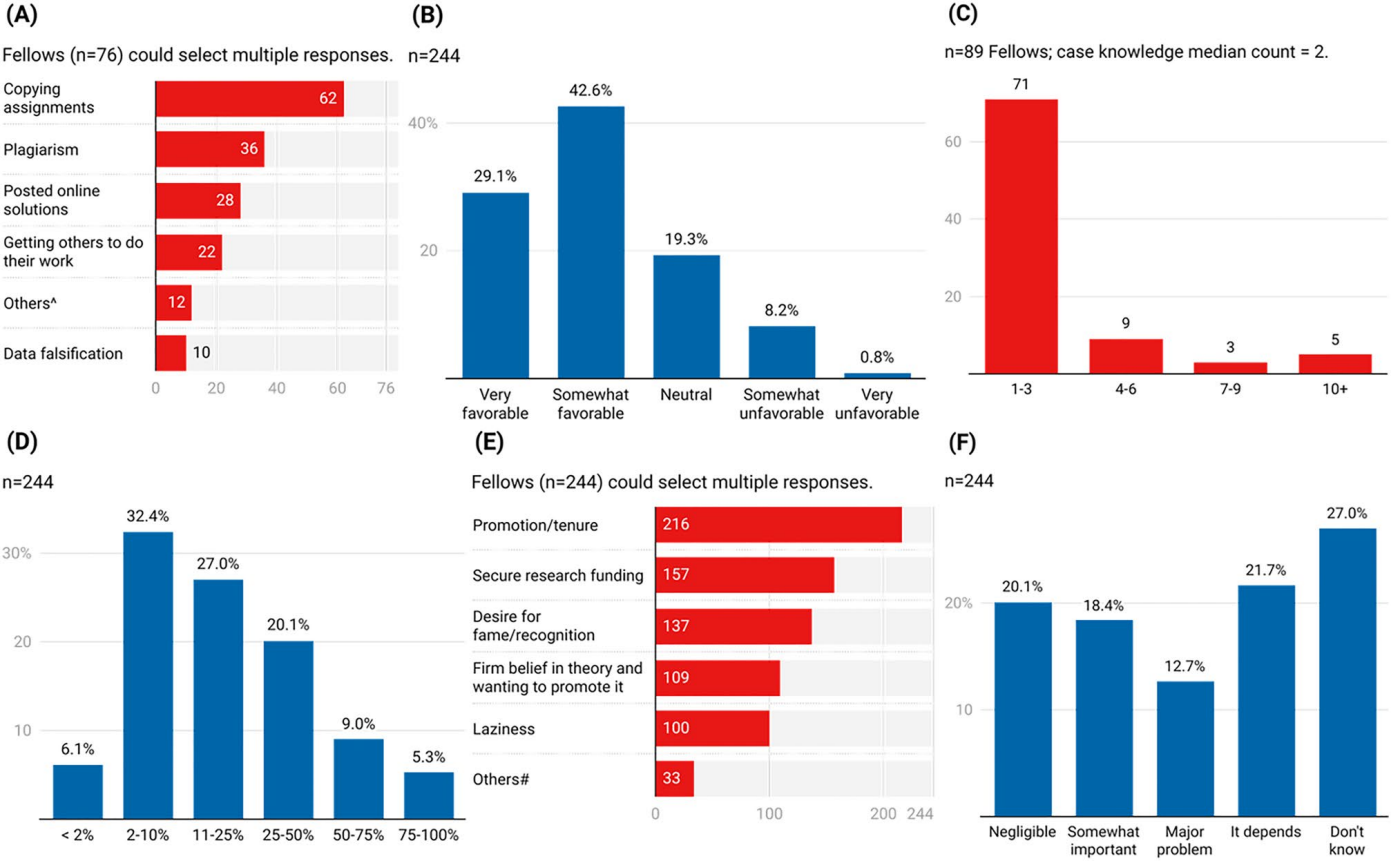
Academic cheating and research misconduct. (**A**) Types of cheating witnessed by NSF Fellows among graduate school peers. (**B**) Perceptions of research integrity practices based on graduate school experiences. (**C**) Histogram of research misconduct case counts Fellows reported direct knowledge of in their fields (original responses listed in Table S6). (**D**) Perceived proportion of researchers who succumb to pressures and commit misconduct at least once in their career. (**E**) Factors that contribute to misconduct or fraud. (**F**) Impact of uncovered fraud on their field. ^^,#^Responses under “Others” listed in Tables S4 and S6, respectively.

Over half (62.3%; n = 152) noted that the level of cheating made them rethink their career choice and the people their field was attracting. The top three most common types of cheating Fellows witnessed their graduate school peers engage in (Fig. 2A) were copying assignments (81.6%), plagiarizing (47.4%), and using online solutions (36.8%). One in five (21.3%; n = 52) individuals acknowledged that academic environments could be made sufficiently perverse due to poor class design, hypercompetitive grading or unfair homework practices, that made cheating justified or acceptable (Table 3). Additionally, 23.4% fellows (n = 57) reported witnessing graduate classes and situations where perverse environments where they felt cheating was necessary or even justified. Despite the above, nearly 9 in 10 fellows (88.9%; n = 217) agreed with the declaration that maintaining their integrity outweighed incentives to cheat (Table 3). Based on their graduate school experiences, 71.7% fellows (n = 175) had a somewhat or very favorable perception of current research integrity practices, while only 9% (n = 22) viewed the policies as somewhat or very unfavorable (Fig. 2B).

The top two reasons Fellows offered for committing academic cheating or considered a motivation for their peers cheating (Table 4) were good grades (e.g., “afraid of bad grades—ashamed of having done so!”) and having less time (e.g., “felt too busy, had to cut corners to get everything done”). In one department, it was asserted that cheating was the norm (i.e., “it [was] unusual if you DON'T have the homework solutions ahead of time”). In another, “getting at least the A or B grade [was] required to continue in the program.” The drive to stay competitive (e.g., “I felt that it was a gray area and that I wanted to have a leg up on my classmates”), the advanced nature of graduate-level classes, and preference to do research over classwork (e.g., “classes are a waste of time, would rather do research”), were less prominent but still notable factors (Table 4) motivating Fellows and their graduate peers to cheat. Altruism (e.g., “I was enjoying working with friends and wanted to help them”) was also mentioned.

**Table 4:** Motivation for academic cheating in graduate school.

(A) Motivations for graduate school peers cheating, per Fellows (n = 76)		(B) Motivations for Fellows themselves cheating (n = 39)	
Motivation	Count (%)	Motivation	Count (%)
Avoid bad grades/failing or get good grades	33 (43.4%)	Time pressure (or heavy workload)	15 (38.5%)
Time pressure (or mismanagement)	21 (27.6%)	Avoid bad grades/failing or get good grades	6 (15.4%)
Disinterest (or laziness)	11 (14.5%)	Stay competitive, high-performing or ahead	5 (12.8%)
Course was too advanced	9 (11.8%)	Accepted way of completing assignments	4 (10.3%)
Preferred research over coursework	8 (10.5%)	Professor did not teach well	3 (7.7%)
Stay competitive, high-performing or ahead	5 (6.6%)	Preferred research over coursework	3 (7.7%)
Faced language barriers as international students	4 (5.3%)	Course was too advanced	2 (5.1%)
Under stress	4 (5.3%)	Desperation or avoid embarrassment	2 (5.1%)
Ignorant on what constitutes cheating	3 (3.9%)	Disinterest	2 (5.1%)
Came from a different academic culture as international students	2 (2.6%)	Ensure I understood the material	2 (5.1%)
Others (1 count each): Stay in the US; Make up for personal deficiencies; Struggling with mental health problems; Ensure they understood the material; Will not get caught	5 (6.6%)	Help friends	2 (5.1%)
		Others (1 count each): Ambiguous expectations around group work; Assignments not representative of knowledge; Class lectures irrelevant to homework; Convenience; Exam sourced from online sources; Ridiculous standards	6 (15.4%)

NSF Fellows’ (A) perceived motivations for graduate peers cheating and (B) motivations for themselves cheating in college and graduate school. Ranked in decreasing order by count.

Fellows used negative personality descriptors (e.g., “mismanagement,” “laziness,” or “knew they could get away with it”) to describe their peers’ cheating at least 11 times but such terms were never used to described their own cheating except to acknowledge disinterest in coursework (n = 3). A distinction was sometimes drawn for circumstances where cheating was perceived as defensible (e.g., “convenient, necessary to proceed forward, and ethically neutral in the long run,” “we were not supposed to use textbooks in class and I thought that was ridiculous,” or “only cheated on homework, never on tests”).

### Research misconduct: awareness, participation and future propensity to commit or report

Only 36.5% (n = 89) of respondents had ever heard of cases of research misconduct (sub-groups: male = 39%, female = 33.9%; 2002–07 cohort = 37%; 2012–17 cohort = 36.4%; CSE 40.2%; CEE = 30.7%). There was no significant association between Fellows’ reporting any knowledge of misconduct cases vis-à-vis their gender, fellowship cohort year or discipline (Tables 2 and S2). A significant association was found between Fellows’ academic stage and their likelihood of ever having heard of research misconduct cases (Table S4); over half of tenured/tenure-track professors (51%) reported knowledge compared to less than one-third of graduate students or non-tenure track professionals (28.4%). Twelve percent (11.9%; n = 29) had first-hand knowledge of misconduct by colleagues in their research group, department or field (median case knowledge count = 2; range = 1–10) (sub-groups: male = 15.3%; female = 8.9%; 2002–07 cohort = 10.9%; 2012–17 cohort = 12.1%; CSE = 14.5%; CEE = 9.9%. There was no significant association between direct knowledge of misconduct among Fellows and their gender, fellowship cohort year or discipline (Fig. 2C and Tables 2 and S2). Four percent (n = 9) confessed to participating in research misconduct (sub-groups: male = 4.2%; female = 3.2%; 2002–2007 cohort = 2.2%; 2012–2017 cohort = 4%; CSE = 5.1%; CEE = 1. There was no significant association between Fellows’ likelihood of committing misconduct and their gender, fellowship cohort year or discipline (Tables 2 and S2) and examples ranged from mild (e.g., non-contributing researchers listed as co-authors) to egregious (i.e., statistical manipulation and data fabrication) (Table S8). Interestingly, 7.4% Fellows (n = 18) were not sure if some of their actions qualified as research misconduct (Table 3).

About six percent of Fellows (6.1%; n = 15) believed that < 2% of all researchers succumb to pressures and commit misconduct at least once in their career (Fig. 2D), whereas 61.4% (n = 150) Fellows felt the proportion was > 10% of all researchers; 5.3% (n = 13) estimated 75–100% researchers commit misconduct at least once. Almost nine out of ten (89%; n = 216) Fellows selected promotion and tenure pressures (Fig. 2E) as the most likely cause for misconduct, followed by funding hyper-competition (64%; n = 157), desire for fame (56%; n = 137), firm belief in one’s theory (45%; n = 109), and laziness (41%; n = 100). One Fellow reported that if the tenure system were changed, “this problem [of misconduct] would be fixed.” Only 12.7% Fellows (n = 31) felt that uncovered fraud had a major impact on the progress of their field, while nearly half picked “It depends” (21.7%) or that they did not know (27%) (Fig. 2F).

While 88.9% Fellows (n = 217) said they would not engage in misconduct (i.e., fabricate or falsify data) to gain funding, win scholarships or publish in high-impact journals, another 10.7% (n = 26) Fellows were unsure, and one NSF Fellow (0.4%) said they would (Table 3). If pressured to engage in research misconduct by an advisor 7.4% Fellows (n = 18) said they would do so, 37.5% (n = 87) were unsure and 56.9% (n = 139) said they would not. Only thirty-one percent (30.7%; n = 75) Fellows said they would report another researcher if they suspected misconduct, whereas 60.7% (n = 148) were not sure, and 8.6% (n = 21) said they would not. A significant association was found between the likelihood of Fellows’ reporting misconduct and their gender, with more men saying they would not report relative to women (Table 3).

### Research misconduct: penalties for scientists found guilty

#### Guilty of distorting the scientific record

The top three recommended punishments were public retractions and corrections of the scientific record, firing or revoking of faculty tenure (e.g., “fire them like any other normal job would do to them”), and a permanent public record of the misconduct (Table 5). Recordkeeping suggestions included (a) databases (i.e., “public index of the guilty”) with researcher names and ORCID (or, Open Researcher and Contributor ID) and (b) universal tags next to all published articles (i.e., “a red flag” signifying the author was “found guilty” of misconduct). A loss of reputation, Fellows reasoned, would negatively impact publishing and reduce grant success as punishment.

**Table 5:** What should happen to researchers found guilty of scientific misconduct that (a) distort the scientific record, (b) waste taxpayer dollars, or (c) harm the public? Top 10 responses, ranked in decreasing order by count.

No	(A) Distort the scientific record	#	(B) Waste taxpayer dollars	#	(C) Harm the public	#
1	Public retraction and correction of the scientific record	85	Revoke funding or awards	50	Bring legal charges or conduct criminal investigation	118
2	Revoke tenure or fire them	61	Ban them from receiving government funding	46	Revoke tenure or fire them	59
3	Permanent public record of misconduct	36	Revoke tenure or fire them	31	Jail time	29
4	Depends	29	Depends	28	Depends	27
5	Ban them from publishing	29	Public retraction and correction of the scientific record	23	Public retraction and correction of the scientific record	23
6	Punishment and disciplinary action by university or professional organizations	19	Flag them and increase scrutiny for future funding and publications	22	Ban them from academia and conducting research	16
7	Ban them from receiving government funding	19	Fine them	20	Ban them from receiving government funding	15
8	Conduct review of all past publications and research	17	Ban them from receiving government funding (some: temporarily)	20	Fine them	13
9	Revoke funding or awards	16	Bring legal charges or conduct criminal investigation	19	Not sure	12
10	Ban them from academia and conducting research	16	Not sure	18	Revoke funding or awards	10

However, there was debate on what constituted distortion of the scientific record. Cherry-picking or reporting only positive results was seen as a “much grayer area,” and “(unfortunately) standard practice in many fields, so it's not clear that there should be a harsh consequence.” One Fellow wrote how “almost every research paper is distorting the results to an extent, because everyone's making a sales pitch.” In contrast, an Earth Sciences Fellow was “surprised” this survey contained questions on research misconduct as this was not an issue at all in their field. Yet another Fellow had witnessed misconduct to the point it was “detrimental to [their] faith in the results of published research across scientific fields” and they left academia post-PhD for industry. Some concerns were also voiced on the unintended consequences of retractions due to misconduct by the PIs on the careers of their graduate students.

#### Guilty of misconduct that wasted taxpayer money

The three recommended punishments for misconduct were = revoking of grant money, permanent ban from receiving government funding, and losing tenure or job (Table 5). However, Fellows were uncertain as to what constitutes “wasting” taxpayer money. Since research does not always work and “plenty of ethical scientific conduct wastes taxpayer dollars,” who will decide “what is a waste of taxpayer dollar <s>.” One Fellow argued “most research areas are a waste,” while another noted, “every project has at least some people who think it's a waste of money.” One Fellow’s perspective on punishing academics for misconduct focused on “if there was willful distortion of science,” which could apply to falsifying data or misrepresenting results to gain funding or committing such acts after receiving grant money. There was also the worry that the “wasting taxpayer dollars” argument could be “politicized in potentially harmful ways.”

In contrast, other Fellows opined that beyond a certain criterion, wasting money on fraudulent work should be “illegal” and that “misconduct is misconduct and penalty should not be different depending on funding source”. Others stated that “There should only be legal consequence if they broke the law,” like embezzlement, and wasting of taxpayer money should be treated as “pretty much like any fraud, graft, or corruption crime” and punishment should be “commensurate with whatever penalties there are for politicians.”

Some perspectives focused on the researcher’s department or university and their obligation to return the funding because the financial waste occurred on their watch and their failure to monitor faculty (“This would make them advocates for good research practices. And their tenure processes are part of this problem”). While one Fellow felt that barring such researchers from getting public grants and preventing them from running a lab seemed “cruel,” another stated, “if I could get my way, I'd also sue them for my tax money back.” Fellows also voiced the view that researchers should be made to “pay it back,” by forfeiture all remaining funds (except salaries of existing employees and graduate students), community service, or repayment of everything out of pocket.

#### Guilty of misconduct that harmed the public

Nearly half of Fellows (48.4%, n = 118) recommended legal charges be brought or a criminal investigation initiated against those guilty of harming the public (Table 5). Revoking of tenure or firing and jail time were the next two most cited penalties. While one Fellow argued that even if a researcher conducted their work unethically, they should not be “held liable for unforeseen repercussions” but investigated purely for misconduct, whereas another felt they should be fired since falsified research, for example, in their area of climate change and infrastructure could lead to poor policies harming the public. One Fellow claimed that the public could only be hurt by policymakers, as they decide “what to do with science results and to measure the impact of those decisions, not scientists.” This is interesting because other Fellows cited the fraudulent and retracted “vaccines cause autism” study^22,23^, and the horrific 40-year experimentation by government physicians on Black men in the Tuskegee syphilis study^24,25^ as exemplar cases of distortion of truth and deliberate public harm by scientists deserving of criminal prosecution and jail time.

### Integrity training and ethical role models

**Figure 3:**
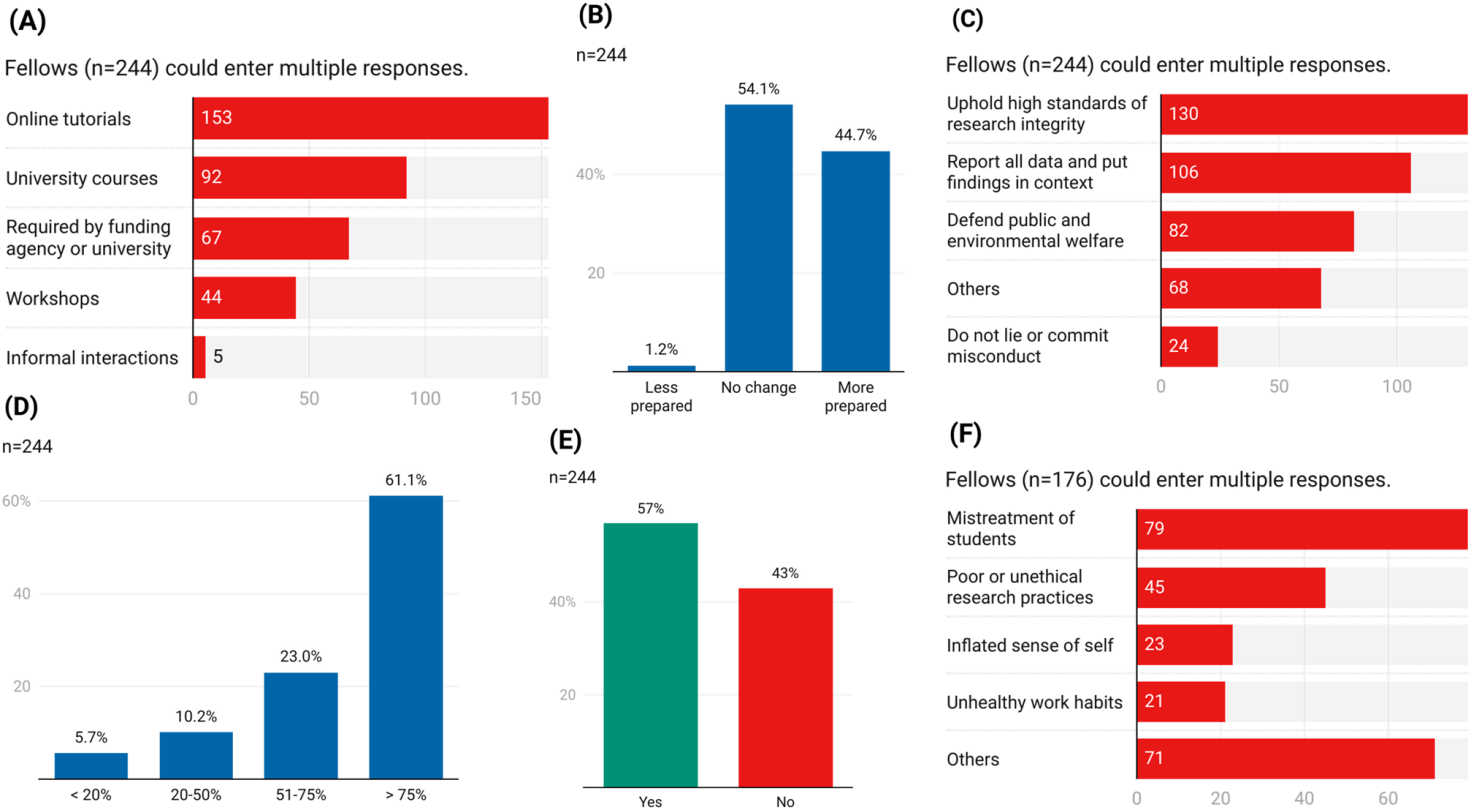
Ethics training and ethical behavior. (**A**) Types of formal ethics training NSF Fellows received. (**B**) Whether these trainings left Fellows prepared to handle ethical issues in graduate school and beyond. (**C**) What does being an ethical scientist or engineer mean to Fellows? (**D**) Percentage of Fellows’ graduate school professors who were good role models. (**E**) Fellows who considered if potential PhD advisors were good role models when choosing graduate schools. (**F**) Top reasons, if any, why Fellows’ advisors were not good role models.

In response to open-ended questions asking to describe their formal integrity trainings, NSF Fellows recalled attending various combinations of online tutorials (62.7%; n = 153) (e.g., Responsible Conduct of Research training from Collaborative Institutional Training Institute [CITI]), university courses at the undergraduate or graduate levels (37.7%; n = 92), and workshops (18%; n = 44) (e.g., graduate school orientation), and mandated by funding agencies or home institutions (27.5%; n = 67) (Fig. 3A). While 44.7% (n = 109) said the trainings left them “more prepared” to deal with ethical issues in graduate school and beyond, over half (54.1%; n = 132) reported the trainings had no effect (Fig. 3B). Fellows believed that ethical scientists and engineers should uphold high standards of research integrity (53.3%; n = 130), report all data and put findings in context (43.4%; n = 106), defend the public or environmental welfare (33.6%; n = 82), and do not lie or commit misconduct (9.8%; n = 24) (Fig. 3C). Other favored character traits (n = 68) included: treat everyone fairly and with respect (n = 12), collaborate or share credit with others (n = 12), and prioritize real scientific progress over mediocre work (n = 9).

Eight-four percent (84.1%; n = 205) Fellows reported that over half of their graduate school professors were good role models, while 5.7% (n = 14) Fellows felt only < 20% of their professors met that standard (Fig. 3D). Over half of the Fellows (57%; n = 139) took into consideration whether potential advisors were good role models when choosing graduate schools (Fig. 3E). The top reasons (n = 176) Fellows’ actual PhD advisors did not turn out to be good role models (Fig. 3F) were (a) mistreatment of students (n = 79; e.g., “Misled students about university policy to delay graduation so they would publish more papers” and “highly critical of graduate student work in a manner that didn't foster that student's training”), (b) poor or unethical research practices (n = 45; e.g., “publishing data of poor quality”, “using creative statistics”, “worked far outside his expertise and didn't know he was ignoring existing work” and “not aware of IRB standards and did not conform to them [putting] graduate students in a tough spot”), (c) an inflated sense of self (n = 23; e.g., “textbook narcissist”), and d) unhealthy work habits (n = 21; e.g., “workaholics and emotionally abusive (but still ethical)”). Less common examples (n = 71) cited: concerned solely about publication count or funding dollars (n = 12), bad or uncaring teaching/mentoring, (n = 12) and being discriminatory (n = 11), primarily sexist (n = 8; e.g., “treated me differently for being female”).

### Lying on this survey

Six (2.5%; n = 6) Fellows confessed to lying in their survey responses, while 40.2% (n = 98) of the remaining group acknowledged they were tempted to lie in response to our survey questions (Tables 6 and Table S3).

**Table 6:** Dishonesty in this survey.

			I was tempted to lie		Total
			Yes	No	
I lied	Yes	6			6 (2.5%)
	No	0	98	140	238 (97.5%)
Total		6 (2.5%)	98 (40.2%)	140 (57.3%)	244 (100%)

Crosstab of NSF Fellows tempted to lie and confessed to actually lying on this survey.

## Discussion

Our national survey compiled academic perceptions and experiences of 244 recipients of the NSF graduate research fellowship that “recognizes and supports outstanding graduate students in NSF-supported STEM disciplines” and has a competitive 15–16% acceptance rate^26,27^. It provides the first ever snapshot of perceptions about academic cheating and research misconduct amongst this high-performing group of researchers. This work summarizes NSF Fellows’ assessments of dominant academic incentives, motivations potentially guiding unethical behavior, and desired professional and legal penalties for serious offenses. The findings cast doubt on the quality and effectiveness of scientific integrity trainings being offered nationwide to engineering graduate students in promoting ethical awareness and behavior. Finally, the survey captures perceptions about the benefits and detriments of an academic career.

### Academic dishonesty, research misconduct and ethics trainings

The 16% cheating rate among NSF Fellows is less than half of that estimated from large, national undergraduate and graduate student surveys^15,28,29^. This discrepancy might be partly explained by the fact that the definition of what constitutes as “cheating” among students is shrinking and the growing rationalization of such behaviors as being sometimes acceptable^15,30–32^. NSF Fellows may also represent a subset of graduate students, who are less likely to cheat in the first place. Moreover, the comparable rates of cheating among men and women^15^ as well as the top reasons for engaging in it—grades and time—are also consistent with prior studies^30,33^.

The research misconduct rates (3.7% for self-reported and 11.9% for direct knowledge of colleagues) are of magnitude similar to those estimated in the most recent global meta-analysis (2.9% and 15.5%, respectively), which relied on 42 surveys (n = 23,228 net participants from 18 countries) conducted over the past three decades^34^.

Factors that Fellows believe majorly contribute to scientific misconduct fit the Fraud Triangle hypothesis for white-collar crime, where external pressures (e.g., for promotion/tenure, funding hyper-competition), opportunity (e.g., desire for fame and recognition), and rationalization (e.g., firm belief in pet theories) result in “a secret violation from a position of trust to commit unethical behavior”^35–37^. Several Fellows echoed recent calls in the literature to prosecute severe research misconduct as a white-collar crime, make investigation reports public and list guilty perpetrators on internet databases to promote transparency and discourage misconduct^38–42^. Personality traits like desire for fame/recognition, narcissism and sociopathy were mentioned as possible factors behind misconduct (Fig. 2E and Table S7). While past research has shown that scoring high on narcissism, psychopathy, and certain Big Five traits (e.g., high extraversion with high IQ) can promote lying, fraud and forgery, or contagion effects spreading to other researchers^43,44^, these results require further investigation among scientists.

While a feared consequence of not punishing dishonest behavior among students is that they could later become “cheating professors”^45^, no systematic studies have directly tested this relationship to our knowledge. However, ~ 20% of Fellows who confessed to academic dishonesty also admitted to or were unsure if they had committed research misconduct (Table S9). Interestingly, while CEE Fellows reported cheating and saw their peers cheat at twice the rate of their CSE counterparts, those in CSE acknowledged higher participation in misconduct and had higher knowledge of colleagues’ misconduct than the former. The emphasis on the First Canon (“to hold paramount the health, safety and welfare of the public”) in the training of CEE Fellows could explain why CEE Fellows perceive themselves as committing misconduct at lower rates than CSE Fellows, but the high self-reported cheating rate among CEE Fellows, while lower than national averages for graduate students^28^, needs further evaluation.

Less than 1 in 3 Fellows believed scientific misconduct to be a somewhat or major problem, compared to nearly half of Americans, who view it as a moderate to very big problem^46^. NSF only started mandating scientific integrity or “Responsible Conduct of Research” training programs starting in 2010^47,48^; over 2200 institutions now offer web-based trainings through the Collaborative Institutional Training Initiative alone^49^. While their effectiveness in reducing scientific misconduct has not yet been evaluated^50^, it is concerning that the trainings reportedly made no difference in ~ 54% Fellows’ ability to handle ethical dilemmas and 7% were unsure if they had ever committed misconduct.

Over one-tenth of Fellows explicitly viewed misconduct on a spectrum ranging from honest mistakes to willful fraud, and believed that professional and legal sanctions (i.e., article corrections and retractions, firing and revoking of tenure and criminal trials) (Table 5) should be commensurate to the severity and frequency of the misconduct. While the scientific community bears collective responsibility to discourage, detect and sanction research misconduct^1^, many academics and institutions do not think such incidents merit serious consideration or investigation^51–54^.

Our survey found NSF Fellows to be astonishingly uninformed as nearly two-thirds had never heard of misconduct cases in their field and this was true for both the older (2002–2007; 63%) and younger (2012–2017; 63.6%) cohorts as well as CSE (59.8%) and CEE (69.3%) Fellows. Chubin contended that the ultimate responsibility to uncover misconduct rests on individual scientists^55^, and indeed, whistleblowers have been the most common way prominent research fraud cases came to light^56^. However, less than one in three Fellows said they would report misconduct and more than half were not sure if they would do anything. This is probably not surprising given that academics usually have no incentive beyond curiosity, self-interest or a sense of duty to investigate research misconduct^57,58^. Moreover, the repercussions of exposing unethical behavior are potentially catastrophic for whistleblowers, as journal articles, grant applications and awards are anonymously reviewed by peers^59^ and severe mental health problems can result from academic shunning and retaliation^60^. On the other hand, if “universities shoulder a major responsibility for exercising control over scholarship misconduct” based on who they hire and promote and how they respond to misconduct allegations^1^, the incentives for departments and universities where unethical professors bring in large amounts of funding can create conflicts of interest and should be considered.

While various policies and protocols have been suggested to reduce cheating and research misconduct (e.g.^41^), efforts should consider both individual motivations and academic pressures^15,61,62^. Pressure to get promotion/tenure was top-ranked by Fellows as possible motivation behind unethical behavior, which is consistent with recent findings on researcher career stage being a predictive factor for journal retractions that mostly result from scientific misconduct^11,63,64^. Integrity training should likely include real world and field-specific case studies and instruction rooted in human nature and organizational psychology, like the TRAGEDIES (i.e., Temptation, Rationalization, Ambition, Group and authority pressure, Entitlement, Deception, Incrementalism, Embarrassment, Stupid systems) and Public-inspired Science models^65,66^, and not driven by compliance alone.

### Dominant academic incentives and ethical research climate

The most emphasized positives of academic careers, i.e., academic freedom, flexible schedule and mentoring opportunities, could explain why 56% of graduate students^67^ and 80% of postdoctoral scholars^68^ still considered academia their career destination of choice, despite an extremely crowded job market and shortening academic career spans^20,69,70^. The intense pressures tied to research grants, publishing, and tenure described by Fellows are also some top reasons given by faculty and graduate students leaving universities following the COVID-19 pandemic^71^. Focus on metrics can also lead to (a) more importance being paid to incremental science instead of novel, transformative topics^72^, (b) misrepresentation and possible distortion of research findings in publications^73^, (c) discounting of scientific contributions by early-career researchers with fluctuating productivity in their initial years making them “more vulnerable to early termination”^74^, (d) preferential retention of young faculty whose productivity may be partially tied to being employed at more-prestigious institutions^75^, and (e) failure to fully capture “research impact” in tenure and promotion practices by not including socially and scientifically relevant outcomes, including transformative community-based research^76^. Moreover, the quantitative metrics arms race may lock researchers on to a “hedonistic treadmill,” where ever higher article counts and funding dollars need to be pursued to sustain an academic career per Goodhart’s Law and possibly maintain one’s self-worth^5,77^.

Fostering ethical cultures i.e., work environments that are supportive of research integrity, is one of 14 core responsibilities of Principal Investigators^78^, but over 1 in 5 Fellows felt they could not discuss wrongdoing in their research groups. Academic advisors arguably influence the academic ethical principles of graduate advisees to a disproportionate degree because if pressured by their advisor to commit misconduct, 2 in 5 fellows either said they would do so or were unsure. “Positive mentorship” has been deemed the “most important factor in completing a STEM degree”^17^ and exhibiting truly ethical leadership by PhD advisors (Fig. 3C) could also help graduate students spot academic temptations, manage ethical dilemmas and avoid questionable practices, thereby contributing to aspired positive and productive research cultures^79^.

Our study has significant limitations. Since women made up 43% (17,577/40,850) of all US doctoral degree recipients in science and engineering in 2021^80^ and the fact that women PhD graduates are over-represented in some STEM fields (e.g., Environmental Engineering [53.2%], Biology [53.8%], and Health and Medical Sciences [71.4%]) and under-represented in others (e.g., Civil and Environmental Engineering [33.3%], Civil Engineering [29.4%], Engineering overall [25.5%], Computer Science and Engineering [20.9–23.6%])^81,82^, responses from our survey cohort (50.8% female) may not be fully representative of STEM academia. The cheating and research misconduct rates are likely underestimates, as is expected with responses to questions of sensitive nature, even in anonymous surveys like ours^5,15^. NSF Fellows are also a group least subject to financial pressures during graduate school due to NSF funding and, therefore, perhaps more likely to accurately describe dominant incentives and external pressures, which may be worse for the typical graduate student. Future research on these topics should survey more representative U.S. academic populations akin to that in The Netherlands^83^ and United Kingdom^84^. This survey dataset could be analyzed using cross correlation matrices for possible inter-relationships between respondents’ attitudes and self-reporting of cheating, misconduct and ethics training compliance, their beliefs on penalties for those found guilty and, more generally, pros and cons of academia. Beyond tracking incidence, research can be designed in conjunction with educational psychologists, moral psychologists, neuroscientists, behavioral economists and legal scholars to: (a) design ethics training and interventions that reduce occurrence of academic dishonesty and research misconduct, (b) isolate institutional and field-specific factors that impact motivation and likelihood of misconduct, (c) study the relationship between individual personality traits vis-à-vis academic cheating and misconduct, and (d) formalize and refine conflicts of interest, penalties and reparation processes for misconduct. Finally, this survey instrument (Text S1) could also be condensed, standardized and administered every 10 years to temporally track trends among NSF Fellows and, more generally, NSF-funded scientists.

## Supplementary Information

Supplementary Information.

## Data Availability

Supporting data are available to bona fide researchers; please contact the corresponding author at sidroy@vt.edu.
