# Supplementary material for: NSF Fellows’ perceptions about incentives, research misconduct, and scientific integrity in STEM academia
Source: Sci Rep. 2023 Apr 7;13:5701. doi: 10.1038/s41598-023-32445-3 (PMC10080524; doi:10.1038/s41598-023-32445-3)
Supplement: Supplementary file 1 — Supplementary Information. [file 41598_2023_32445_MOESM1_ESM.pdf]

Supplementary Information for

NSF Fellows’ perceptions about incentives, research misconduct, and scientific integrity in STEM academia

Siddhartha Roy and Marc A. Edwards

Siddhartha Roy
Email: sidroy@vt.edu

SI Table of Contents

Table S1. Survey Response Rates for NSF Fellows surveyed between February 18 and May 02 2019.
Table S2. NSF Graduate Research Fellowship award discipline sub-categories.
Table S3. Cross-tabulation of NSF Fellows’ disciplines vis-à-vis responses to academic dishonesty and research misconduct questions.
Table S4. Cross-tabulation of NSF Fellows’ academic stage vis-à-vis responses to academic dishonesty and research misconduct questions.
Table S5. Types of academic cheating listed under “Others” in Figure 2A.
Table S6. Raw data for research misconduct cases you have heard of in the last five years.
Table S7. All factors contributing to research misconduct/fraud categorized under “Others” in Figure 2E and reproduced as-is.
Table S8. Examples of research misconduct committed or participated in.
Table S9. Cross-tabulation of NSF Fellows who confessed to research misconduct and academic dishonesty.
Text S1. Survey Instrument.

Table S1. Survey Response Rates for NSF Fellows surveyed between February 18 and May 02 2019.

Graduate studies field \ Six-year cohort		2002-07	2012-17	All	Response Rate^
Civil and Environmental Engineering	Total awarded	226	368	594	30.2%
	Survey email delivered*	113	255	368	
	Completed survey	22	89	**111**	
Computer Science and Engineering	Total awarded	447	621	1068	18.7%
	Survey email delivered*	238	472	710	
	Completed survey	26	107	**133**	
**Total survey respondents**		**46**	**198**	**244**	**22.6%**

* i.e., active email address found and email sent did not bounce/fail.

^ response rate = count of surveys completed ÷ count of survey emails delivered.

Table S2. NSF Graduate Research Fellowship award discipline sub-categories.

**Civil and Environmental Engineering**
Civil Engineering<br>Environmental Engineering<br>Water Resources Engineering
**Computer Science and Engineering**
Computer Engineering
Computer Science<br>Algorithms and Theoretical Foundations<br>Artificial Intelligence/Robotics/Computer Vision/Expert Systems/H,<br>Communication and Information Theory,<br>Computer Architecture and Grids,<br>Computer and Information Security,<br>Computer Systems Design/Signal Processing,<br>Computer Security and Privacy,<br>Database Systems,<br>Data Mining and Information Retrieval<br>Database Information Retrieval and Web Search,<br>Formal Methods, Verification and Programming Languages,<br>Graphics and Visualization,<br>Human Computer Interaction,<br>Informatics/Bioinformatics,<br>Information and Systems,<br>Information Security and Assurance,<br>Information Technology and Organizations<br>Languages and Systems,<br>Machine Learning/Data Science,<br>Natural Language Processing,<br>Networks and Communications,<br>Operating Systems and Middleware,<br>Scientific Computing and Informatics,<br>Software Engineering,<br>Theory/Theoretical Foundations,<br>Others:<br>Medical Imaging Science, Parallel Computing, Quantum Computing, SYS DYN, Nanotechnology/Molecular Computation, Pervasive Computing, Artificial Life and Ecologies, Computations Organizations and Society, Language and Information Technologies, Bioinformatics, Computational Biology, High Performance Computing, Assistive Technology, Technical and Social Behavior, Multi-Agent Systems, Cognitive Science, Formal Methods, Social Sensing, Brain-Computing Interfacing, Biocomputation, Human Robot Interaction.

Note: Award categories evolved during 2002-17, with addition of new categories and merging of others.

Table S3. Cross-tabulation of NSF Fellows' disciplines vis-à-vis responses to academic dishonesty and research misconduct questions. Data available for 218 of 244 Fellows.

		**Total**	**Unknown***	**CSE** n (%)	**CEE** n (%)	**p-value** (CEE vs. CSE)
Response data available for (count)		244	26*	117 (100)	101 (100)	N/A
Personally cheated	Yes	39	2	13 (11.1)	24 (23.8)	***0.018****
	No	205	24	104 (88.9)	77 (76.2)	
Saw peers cheating	Yes	76	9	27 (23.1)	40 (39.6)	***0.012****
	No	168	17	90 (76.9)	61 (60.4)	
Has heard of research misconduct	Yes	89	11	47 (40.2)	31 (30.7)	>0.05**
	No	155	15	70 (59.8)	70 (69.3)	
Direct knowledge of research misconduct	Yes	29	2	17 (14.5)	10 (9.9)	>0.05**
	No	215	24	100 (85.5)	91 (90.1)	
Personally committed research misconduct	Yes	9	2	6 (5.1)	1 (1)	>0.05***
	No	217	24	101 (86.3)	92 (91.1)	
	Not sure	18	0	10 (8.5)	8 (7.9)	
Lied on this survey	Yes	6	1	5	0	>0.05**
	No	238	25	112	101	
Tempted to lie (total n=238)	Yes	98	14	43	41	>0.05**
	No	140	11	69	60	

* 26 unknown = 10 CEE + 16 CSE.
** Fisher's Exact Test
*** Chi-Square Test

Table S4. Cross-tabulation of NSF Fellows' academic stage vis-à-vis responses to academic dishonesty and research misconduct questions.

		**Total**	**Tenure-track or Tenured** n (%)	**Graduate student or Non-Tenure-track** n (%)	**Non-academic** n (%)	**p-value***
Count		244	49 (100)	123 (100)	72 (100)	N/A
Personally cheated	Yes	39	8 (16.3)	18 (14.6)	13 (18.1)	p>0.05**
	No	205	41 (83.7)	105 (85.4)	59 (81.9)	
Saw peers cheating	Yes	76	10 (20.4)	41 (33.3)	25 (34.7)	p>0.05**
	No	168	39 (79.6)	82 (66.7)	47 (65.3)	
Has heard of research misconduct	Yes	89	25 (51)	35 (28.4)	29 (40.3)	***p=0.015*** X^2=8.3376***
	No	155	24 (49)	88 (71.6)	43 (59.7)	
Direct knowledge of research misconduct	Yes	29	7 (14.3)	12 (9.8)	10 (13.9)	p>0.05**
	No	215	42 (85.7)	111 (90.2)	62 (86.1)	
Personally committed research misconduct	Yes	9	3 (6.1)	5 (4.1)	1 (1.4)	p>0.05**
	No	217	45 (91.8)	107 (87)	65 (90.3)	
	Not sure	18	1 (2)	11 (8.9)	6 (8.3)	
Lied on this survey	Yes	6	0 (0)	2 (1.6)	4 (5.6)	N/A
	No	238	49 (100)	121 (9.8)	68 (94.4)	
Tempted to lie (total n=238)	Yes	98	15 (30.6)	58 (47.9)	25 (36.8)	p>0.05**
	No	140	34 (69.4)	63 (52.1)	43 (63.2)	

Percentages may not add up to 100 because of rounding.
* Tenure-track/Tenured vs. Graduate students/Non-Tenure-track vs. Non-academic positions
** Chi-Square Test

Table S5. Types of academic cheating listed under "Others" in Figure 2A.

Type of practice	Frequency
Cheating in exams, including sharing answers or using unpermitted resources. (e.g., "claiming a cell phone was a translator and texting answers [during] exams")	4
Groups collaborating on work/assignments/take-home exams meant for individuals (e.g., after signing honor codes explicitly promising to not collaborate)	3
Use of cellphones during exams	2
Reviewing homework from previous years	1
Use of unauthorized reference materials	1
Intentional data misrepresentation	1

Table S6. Raw data* for research misconduct cases you have heard of in the last five years (Figure 2C).

Open-ended Response	Recorded response	Count
NA	0	1
1	1	24
2	2	26
2 major, many minor	2	1
2 or 3	3	1
3	3	16
A few	3	2
a few, maybe 2-3	3	1
5	5	6
5 or 6	6	1
5+	6	1
6	6	1
a handful of papers (4-7) I have seen retracted	7	1
Many	8	1
Several – ethics around data science especially	8	1
1-10 times	10	1
10	10	2
5-10	10	1
No idea; lots	10	1
**Total fellows count**		**89**
**Median case knowledge count**	**2**	

*Methodology of converting non-numerical entries into actual counts: If Fellows offered a range, the maximum value was chosen. If Fellows used a purely explanatory word or phrase, the following convention was used: few = 3, several or many = 8, lots = 10.

Table S7. All factors contributing to research misconduct/fraud categorized under "Others" in Figure 2E and reproduced as-is.

• "The end justifies the means" logic
• A culture in which misconduct is the norm.
• Afraid to have research fail
• Sometimes, it can be extremely difficult to follow IRB procedures - it's not laziness, it's actually difficult to figure out what they want us to do.
• Concern that inconclusive results are not publishable
• For [international] student: remaining in US
• Not working closely enough with their graduate students who may be pressured to fabricate data in order to graduate, get a job, etc
• Influence of advisor (pressure)
• Influence of advisor (appeasement)
• Wanting to get publication out
• Greed (grant mis-spending)
• Sociopathic personalities
• Narcissism/Ego/Inability to believe you are wrong
• Myth of exceptionalism
• Carelessness
• Poor training
• Ignorance of what constitutes misconduct
• Lack of ethics training
• Some people have different ethical standards/ morals (even if they're objectively wrong)
• Different interpretation of misconduct/fraud (e.g., when reusing existing data might not be ethical)
• Lack of understanding that the action is misconduct/fraud
• Lack of realization that [they are] being unethical
• Insufficient attention to detail
• Lack of education/experience in doing properly controlled studies
• Lack of sufficient oversight by senior researchers combined with lack of training of junior researchers
• Naiveté/lack of knowledge
• Incompetence
• incompetence (when just did the eval wrong), self-delusion (persuaded themselves it was their work, not stolen)
• Lazy, stupid, irresponsible, immoral
• Not wanting a lot of work on a project to have led to confusing or negative results
• Industry research misconduct is to increase profits
• Wanting to support a particular cause
• Sexual desires

Table S8. Examples of research misconduct committed or participated in.

1.	Inaccurate reporting of methods used to analyze data
2.	Modified experimental results to look more favorable
3.	Skewed data with creative statistics
4.	Data falsification
5.	Published old data supporting old hypotheses when new data showed different trends
6.	Advisor added authors to papers who did not contribute
7.	Advisor added authors to paper who did not contribute so that the latter would pay conference fees
8.	Found error in an experiment but did not fix
9.	Caught it on an artifact evaluation committee

Table S9. Cross-tabulation of NSF Fellows who confessed to research misconduct and academic dishonesty.

		**Research misconduct**			
		No	Not sure	Yes	All
**Academic dishonesty**	No	186	13	6	205
	Yes	31	5	3	39
	All	217	18	9	244

Text S1. Survey Instrument

Start of Block: Part 0 Electronic Consent

Hello,

You are invited to participate in this research study on perverse incentives in research academia because you are/have been an NSF Graduate Research Fellow. This research study is being conducted by Virginia Tech researchers Drs. Marc Edwards [(540) 231-7236 / edwardsm@vt.edu] and Siddhartha Roy [(540) 521-6193 / sidroy@vt.edu] as a follow-up to their research paper "Academic Research in the 21st Century: Maintaining Scientific Integrity in a Climate of Perverse Incentives and Hypercompetition."

The survey questions will focus on undergraduate and graduate school experiences, with an emphasis on perceived academic pressures, the issue of integrity in conducting research, temptations to commit misconduct, and ethics training. The survey should take **less than 30 minutes to complete** and you will receive a $25 Amazon.com gift card within 72 hours of completion. The gift card code will be emailed directly to you from Amazon.

Your participation in this research study is voluntary. You may choose not to participate. If you decide to participate in this research survey, you may withdraw at any time. Your responses will be confidential. Furthermore, the "Anonymize Response" setting is active for this survey that ensures Qualtrics will "NOT record any personally identifiable information and remove contact association". The results of this study will be used for scholarly purposes only, including publishing peer-reviewed journal articles and presenting at conferences.

Should you have any questions about this study, you may contact one of the research investigators whose contact information is included at the beginning of this document. This research has been reviewed according to Virginia Tech IRB procedures for research involving human subjects. Should you have any questions or concerns about the study's conduct or your rights as a research subject, or need to report a research-

related injury or event, you may contact the Virginia Tech Institutional Review Board at irb@vt.edu or (540) 231-3732.

ELECTRONIC CONSENT: Please select your choice below.

Clicking on the "agree" button below indicates that:

- you were/are an NSF Graduate Research Fellow
- you have read the above information
- you voluntarily agree to participate

If you do not wish to participate in the research study, please decline participation by clicking on the "disagree" button.

○ Agree (1)

○ Disagree (2)

End of Block: Part 0 Electronic Consent

Start of Block: Part 1 Background

Q1
When did you receive your NSF Fellowship?

○ 2012-2017 (1)

○ 2002-2007 (2)

Q2 What degree are you currently pursuing?

○ MS (1)

○ PhD (after obtaining an MS) (2)

○ Direct PhD (3)

○ Working (Graduated with an MS) (4)

○ Working (Graduated with a PhD) (5)

Q3 Which option best describes your current job profile?

- ○ Academia; Tenure-track/Tenured (1)
- ○ Academia; Postdoc/Researcher/Non-tenured (2)
- ○ Industry/Consulting (3)
- ○ Nonprofit/Government (4)
- ○ Research Laboratory (5)
- ○ Other. Please state: (6) ____

Q4 What was your undergraduate major?

- ○ Civil/Environmental Engineering (1)
- ○ Chemical/Mining/Metallurgy Engineering (2)
- ○ Mechanical Engineering (3)
- ○ Computer Science (4)
- ○ Electrical/Computer Engineering (5)
- ○ Other. Please state: (6) ____

Q5 Please enter your gender in full. (e.g. enter Female and not F):

____

Q6 Have you received any formal academic integrity/ethics training (required by NSF, your university/department, etc.)?

○ Yes (1)

○ No (2)

End of Block: Part 1 Background

Start of Block: Part 2 Cheating

Q1 Have you ever seen fellow students cheat (in graduate school classes)?

○ Yes (1)

○ No (2)

Skip To: Q2 If Q1 = 2

Q1A What type of cheating practices did you personally witness in classes (can select multiple options)?

▢ Copying assignments (1)

▢ Posted online solutions (2)

▢ Getting others to do your work (3)

▢ Plagiarism (4)

▢ Data falsification (5)

▢ Others. Please state: (6)
____

Q1B What was their primary motivation, if you were told or had to guess?

____

____

____

____

____

Q1C Is there a perception that cheating is sometimes acceptable or even necessary for students (due to the workload, or because others are doing it)?

○ Yes (1)

○ No (2)

Q2 Have you personally cheated (in college and/or in graduate school)?

- ○ Yes (1)
- ○ No (2)

Display This Question:
If Q2 = 1

Q2A If yes, can you give the primary reason why you did so?

____

Q3 Did you ever feel that maintaining your integrity outweighs any prevalent incentives to not cheat (possibly lower grade, extra time spent actually doing the work that you could have copied, etc.) in class?

- ○ Yes (1)
- ○ No (2)

Q4 Were there classes/situations, in which an environment was created, where it could be argued that cheating was necessary or justified?

- ○ Yes (1)
- ○ No (2)

Q5 Did you ever think that the level of cheating you witnessed or engaged in, was such that you thought twice about your career choice and the type of people the profession was attracting?

- ○ Yes (1)
- ○ No (2)

Q6 Do you believe it is possible, through poor design of classes or unfair homework or hypercompetitive grading structures, to create an environment where cheating in classes would be justified or acceptable?

- ○ Yes (1)
- ○ No (2)

End of Block: Part 2 Cheating

Start of Block: Part 3 Research Practices

Q1 Research Misconduct (as described by the NSF) includes willful fabrication, falsification, plagiarism, and other questionable practices. For the purposes of this survey, please consider this definition when answering the following questions. Are you considering or already pursuing a research career (in a lab or as faculty)?

- ◯ Yes (1)
- ◯ No (2)

Q2 Based on your research experiences in graduate school, what is your perception of research integrity practices?

- ◯ Very favorable (1)
- ◯ Somewhat favorable (2)
- ◯ Neutral (3)
- ◯ Somewhat unfavorable (4)
- ◯ Very unfavorable (5)

Q3 What, do you believe, are the pros and cons of getting a job in academia – list at least the two most important “pros” and two most important “cons”?

- ◯ Pro 1 (1) ____
- ◯ Pro 2 (2) ____
- ◯ Other Pros (3) ____
- ◯ Con 1 (4) ____
- ◯ Con 2 (5) ____
- ◯ Other Cons (6) ____

Q4 Do you have **direct** knowledge of scientific misconduct in your research group/department/field in the past 5 years?

- ○ Yes (1)
- ○ No (2)

Q4A Have you heard of research misconduct cases in your field in the past 5 years?

- ○ Yes. If so, how many (1) ____________________
- ○ No (2)

Q5 Have **you** participated in research involving misconduct in the past 5 years (even if you did not commit them yourself)?

- ○ Yes. If so, what kind: (1) ____________________
- ○ No (2)
- ○ Not sure (3)

Q6 In your opinion, what factors contribute to a researcher committing misconduct/fraud (pick one or more)?

- ☐ Job pressures/Promotion/Tenure (1)
- ☐ Secure research funding (2)
- ☐ Desire for fame/Recognition (3)
- ☐ Firm belief in a theory and wanting to promote it (4)
- ☐ Laziness (5)
- ☐ Others. Please list: (6) ____________________

Q7 If you felt pressured by your academic advisor/mentor to engage in scientific misconduct (“everyone is doing it this way”, “it is okay to throw out bad data”, or publishing results before you are personally confident), would you do it?

- Yes (1)
- No (2)
- I don't know (3)

Q8 If fabricating and/or falsifying data helped increase your chances to get research funding, scholarship money or publication in a high impact journal, would you do it?

- Yes (1)
- No (2)
- I don't know (3)

Q9 If you suspected a researcher of engaging in misconduct, would you report it?

- Yes (1)
- No (2)
- I don't know (3)

Q10A What should happen to researchers found guilty of scientific misconduct that **distort the scientific record**

____

Q10B What should happen to researchers found guilty of scientific misconduct that **waste taxpayer dollars**

____

Q10C What should happen to researchers found guilty of scientific misconduct that **harm the public**

____

Q11 In your opinion, what is the impact of uncovered fraud in the progress of your field?

○ Negligible (1)

○ Somewhat important (2)

○ Major problem (3)

○ It depends (4)

○ I don’t know (5)

Q12 What criteria do you use to mentally evaluate peers/academics in your field? Rank in order of decreasing importance, with #1 the most important factor and #6 the least important.
______ No. of published articles and citations (1)
______ Publishing in prestigious journals (2)
______ H-index (3)
______ Total funding dollars (4)
______ Scientific advancement of the field (5)
______ Social impact of their work (6)

Q12B Is there another factor that you consider more important in evaluating peers/academics? Please state:
____

Q13 Do you judge yourself using the same metrics as you do your peers?

○ Yes (1)

○ No (2)

Q14 What percentage of your professors (teachers/advisors) do you consider to have been good “ethical models” for yourself (in graduate school)?

- ○ < 20% (1)
- ○ 20-50% (2)
- ○ 51-75% (3)
- ○ > 75% (4)

Q14B Identify the most important reason (if any) advisors were not good role models:

____

Q15 Do you feel empowered to raise questions about wrongdoing (however big or small) in your research group? Example: by not raising your voice/doing the right thing about issues affecting research integrity.

- ○ Yes (1)
- ○ No (2)

Q16 Is “doing the right thing” even though it might negatively impact your career or how you are viewed by peers, something you feel strongly about?

- ○ Yes (1)
- ○ No (2)
- ○ I don't care (3)

Q17 How would you rate science and engineering, compared to your perception of other professions, in terms of actual integrity and serving the public good?

____

Q18 According to a survey expert, people falsify information on surveys all the time. Did you answer falsely to any of the questions posed above?

○ Yes (1)

○ No (2)

Display This Question:
If Q18 = 2

Q18B Were you tempted to do so (answer falsely to the questions posed) even though this is an anonymous survey?

○ Yes (1)

○ No (2)

End of Block: Part 3 Research Practices

Start of Block: Part 4 Ethical Training and Perceptions

Q1 Explain the type of formal academic integrity/ethics training (in class, required reading, online tutorial, workshop, etc.) you received.

____

Q1B Were there examples of research misconduct and unethical practices in your training, such as cheating during the instructional modules (i.e., searching the internet during exams that were supposed to test current knowledge)? Please list.

____

Q1C If so, did that affect your perception of how important it was considered?

____

Q1D Do you think you are more prepared or less prepared, to deal with ethical issues in graduate school and beyond, as a result of the training?

- ◯ More prepared (1)
- ◯ No change (2)
- ◯ Less prepared (3)

Q1E Would you rather not know about, or not think about, the prevalence of unethical practices in your profession?

____

Q2 What does being an "ethical scientist/engineer" mean to you?

____

Q3 If you had to guess, what percent of researchers succumb to pressures, and engage in some form of scientific misconduct at some point in their career?

- < 2% (1)
- 2-10% (2)
- 11-25% (3)
- 25-50% (4)
- 50-75% (5)
- 75-100% (6)

Q4 When researching academic PhD advisors for graduate school, what were the most important factors you took into account?

____

Q4B Did you strongly consider whether they (potential PhD advisors) were good role models?

- Yes (1)
- No (2)

End of Block: Part 4 Ethical Training and Perceptions

Start of Block: Part 5 Role of Scientists and Engineers in Society

Q1A Do you think scientific research ideally is (or should be about) truth-seeking?

- ○ Yes (1)
- ○ Sometimes (2)
- ○ No (3)

Q1B Do you think scientific research ideally is (or should also be about) “service to humanity”?

- ○ Yes (1)
- ○ Sometimes (2)
- ○ No (3)

Q2 In practice, do you think scientific research you have witnessed, has been in “service to humanity” or “truth-seeking”, or have you wondered if it is primarily about self-advancement and/or climbing an academic pyramid?

- ○ Yes (1)
- ○ Sometimes (2)
- ○ No (3)

Q3 Is there anything else you wish to share on these subjects (please do not provide your name or institution)?

____

End of Block: Part 5 Role of Scientists and Engineers in Society

Start of Block: Part 6 Compensation

We thank you for your participation. Your $25 Amazon.com gift card will be directly sent to your email address in the next 72 business hours. If you do not receive your code, please send an email to sidroy@vt.edu.

End of Block: Part 6 Compensation
